# Advancing understanding of the mechanisms of antipsychotic-associated cognitive impairment to minimise harm: a call to action

**DOI:** 10.1038/s41380-024-02503-x

**Published:** 2024-03-07

**Authors:** Kelly Allott, Sidhant Chopra, Jack Rogers, Maria Regina Dauvermann, Scott Richard Clark

**Affiliations:** 1 Orygen, Parkville, VIC Australia; 2 Centre for Youth Mental Health, The University of Melbourne, Parkville, VIC Australia; 3 Department of Psychology, Yale University, New Haven, CT USA; 4 Institute for Mental Health, University of Birmingham, Birmingham, UK; 5 University of Adelaide, Discipline of Psychiatry, Adelaide, SA Australia; 6 Basil Hetzel Institute, Woodville South, SA Australia

**Keywords:** Molecular biology, Schizophrenia, Biochemistry

The discovery in the 1950s that antipsychotic medication could dramatically improve the positive (and in some cases negative) symptoms of severe psychotic disorders led to antipsychotics becoming the first-line treatment for acute recovery, with maintenance for at least one to two years recommended for relapse prevention. Yet, antipsychotic medications do not usually improve the symptoms that underpin poor functional outcome, such as cognitive impairment. Clinically significant cognitive impairment is a hallmark of psychotic illness and is already present prior to the introduction of antipsychotics [1]. Whilst variable, the bulk of evidence shows that antipsychotic effects on general cognitive functioning are *at best* mildly positive and that these positive effects can mostly be explained by cognitive test practice effects, or atypical antipsychotics being less cognitively impairing than typical antipsychotics [2, 3]. A growing body of more recent literature suggests that antipsychotic medication may in fact worsen cognitive functioning, including specific domains such as verbal learning and memory as well as composite functioning [4, 5]. Subjective cognitive impairment, particularly cognitive slowing, is commonly reported by people taking antipsychotics. Naturalistic studies have shown higher cumulative antipsychotic exposure to be associated with poorer cognitive functioning [4], although these findings may reflect confounding by indication. Our recent triple-blind randomised controlled trial compared the effects of risperidone/paliperidone versus placebo on cognitive functioning over the first six months of treatment for first-episode psychosis [5]. A healthy control group not taking placebo or medication was also recruited. We found that in several cognitive domains the stability or improvement observed was similar across the three groups, suggesting improvements were in fact typical and not related to illness or medication [5]. However, a significant interaction was observed for verbal learning and memory, where the healthy control and placebo groups improved, but the risperidone/paliperidone group declined in performance. The effect sizes were moderate to large [5]. At the same time, there has been a rise in the number of randomised controlled trials comparing antipsychotic *dose reduction* with antipsychotic maintenance to evaluate their risk: benefit profile across a range of outcomes not limited to relapse. Cognition is a common outcome of these studies, where preliminary evidence suggests that medically guided dose reduction may be associated with superior cognitive outcomes, including in processing speed and global cognitive function (see Table 1).

**Table 1:** Randomised controlled trials comparing antipsychotic dose reduction with maintenance with cognition as an outcome.

Study	Country6	Psychosis sample	Sample size	Dose reduction period	Primary endpoint	Cognitive outcomes
Faber et al. [17]	Netherlands	First-episode psychosis	42	4 weeks	5 months	+ Processing speed; verbal fluency = Attention; working memory; learning and memory; motor function
Hori et al. [18]	Japan	Long-term schizophrenia	39	12 weeks	5 months	+ Processing speed; attention = Verbal memory; working memory; motor function; verbal fluency; executive function; global cognition
Takeuchi et al. [19]	Japan	Schizophrenia	61	24 weeks	6 months	+ Global cognition; immediate memory; language = Visuospatial; attention; delayed memory
Zhou et al. [20]	China	Schizophrenia	75	12 weeks	12 months	+ Global cognition; processing speed; working memory = Attention; learning; executive function; social cognition
Stürup et al. [21]	Denmark	First-episode schizophrenia	29	12 months	12 months	+ Global cognition

+ indicates that outcomes were superior in the dose reduction group, = indicates the dose reduction and maintenance groups did not differ.

Antipsychotic medications encompass a wide variety of compounds, sharing a common mechanism - antagonism of the dopamine D_2_ receptor. Atypical second-generation antipsychotics additionally antagonise serotonin 5-HT_2_ receptors, and third generation compounds exhibit preferential binding to D_3_ and partial D_2_ receptor antagonism. It is widely accepted that the modulation of these neurotransmitter systems represents the primary mechanism behind reduction of positive symptoms, in support of the dopamine hypothesis. However, the sequalae of this modulation on cognition and variance by antipsychotic class remains understudied. Additional proposed mechanisms of antipsychotic effects on cognition include neuromodulation of excitatory (glutamatergic), inhibitory (γ-Aminobutyric acid) and anticholinergic systems, which impact on inflammatory processes and may be related to side-effects, such as anticholinergic burden, motor symptoms, and sedation. To optimise antipsychotic selection and treatment response with a view to minimising iatrogenic cognitive effects and maximising benefits and prognosis, there is a pressing need to increase our mechanistic understanding of how antipsychotics may alter cognition through systematic investigation.

## Effects of dopaminergic modulation on cognitive function

Dopamine modulation subserves multiple aspects of cognition, especially through the regulation of brain circuits connecting the pre-frontal cortex and striatum via the dopaminergic mesocortical and mesolimbic pathways among others. Previous work from our group has shown that even short-term use of antipsychotics can cause prominent changes in both brain structure and function within cortico-striatal regions [6]. Positron emission tomography (PET) imaging studies demonstrate a hyperbolic association between antipsychotic dose and D_2_ occupancy, where increasing doses are associated with incrementally smaller impacts on D_2_ occupancy, yet adverse effects, including cognitive effects, appear to worsen [7]. Similarly, plasma antipsychotic concentrations corresponding to brain D_2_ receptor occupancy above 70% are associated with disproportionately poorer cognitive outcomes [8]. Open label studies involving antipsychotic-naïve first-episode individuals have shown risperidone (a strong dopamine antagonist) was associated with a decline in cognitive functioning over 12-weeks, with a significant negative correlation observed between extrastriatal D_2/3_ occupancy and cognitive performance [9]. Therefore, the effects of antipsychotics on learning, memory and other executive functions may arise due to prolonged antagonism at D_2_ receptors [8], with a gradual reduction in dose potentially leading to normalisation of dopamine-mediated functional brain circuits underlying higher-order cognitive processes.

## Effects of anticholinergic modulation on cognitive function

While consistent evidence has implicated increased striatal presynaptic dopamine synthesis as a primary illness mechanism underlying psychosis, the neural changes associated with antipsychotic use are not exclusively localised to dopaminergic pathways, but rather may reflect widespread functional brain changes in thalamo-cortical circuits subserving higher-order cognitive processes [6]. Antipsychotic medications (and other psychotropic medications) vary in their degree of antagonism of acetylcholine receptors; a risk factor known as anticholinergic burden. The major cholinergic pathways of the brain originate in the basal forebrain, brainstem, and striatum, innervating all cortical and subcortical regions. Anticholinergic burden from psychotropic (and non-psychotropic) medication is often related to cognitive impairment, with exposure to anticholinergic drugs including antipsychotics being associated with significantly increased risk for dementia [10]. In individuals with psychosis, higher anticholinergic burden is associated with worse performance in multiple cognitive domains, including attention, verbal learning and memory, and working memory, even after accounting for confounds such as medication dose and illness severity [11]. The blockade of muscarinic cholinergic receptors by second-generation antipsychotics may contribute to this cognitive impairment. Conversely, selective muscarinic M_1_/M_4_ agonism is associated with improved negative (and potentially cognitive) symptoms in some patients [12].

## Effects of glutamatergic modulation and neuroinflammation on cognitive function

There is emerging evidence for a complex interaction between inflammation, oxidative stress, dopamine transmission, synaptic plasticity and glutamate transmission including hypofunction of N-methyl-D-aspartate receptors (NMDAR), which lie at the core of pathology in schizophrenia [13] and associated cognitive dysfunction. For individuals with established schizophrenia, but not first-episode psychosis, risperidone appears to decrease levels of proinflammatory cytokines, such as IL-6, TNF-α, IL-2 and IL-1β, but has no significant effect on IFN-γ [14]. In contrast, for clozapine, pro-inflammatory effects can be observed early in treatment, but overall, no significant effects on IL-6 and TNF-α are found [14]. However, there are conflicting findings and further research is needed in this evolving field. Inflammation can also be linked to blood antioxidant levels (reduced) and markers of oxidative stress (increased) in those at high risk of psychosis and in untreated first-episode psychosis, but these levels normalise with long-term antipsychotic treatment [15]. Cytokines such as IFN-γ or TNF-α can also induce hypofunction in NMDAR by increasing the synthesis of kynurenic acid which blocks the NMDAR glycine binding site, but is reversible by clozapine [13]. Thus, the balance of positive and negative cognitive effects associated with antipsychotic neuromodulation must be considered. We advocate that further investigation into the complex neuroinflammatory effects of antipsychotics on cognition, which vary between drugs and across the stage and duration of illness and treatment, is imperative.

## Impact of side-effects on cognitive function

The effects of antipsychotic neurotransmitter modulation on brain circuits subserving cognition, may also manifest as side-effects such as sedation, movement disorders (e.g., extrapyramidal effects), amotivation, and blurred vision, which are relatively common [16]. Side-effects can greatly influence or impair cognitive functioning or hamper performance on cognitive tasks and have further negative consequences for functional outcome, personal recovery, or quality of life. A further common consequence of antipsychotic side-effects, including cognitive effects is that people stop taking their medication without medical guidance. This potentially leads to unintended adverse outcomes, such as relapse and hospitalisation. Antipsychotic-related side-effects are rarely examined as a mechanism of cognitive impairment in psychotic disorders and require more systematic investigation so that treatment response can be optimised.

## Progressing the field through mechanistic studies

**Fig. 1:**
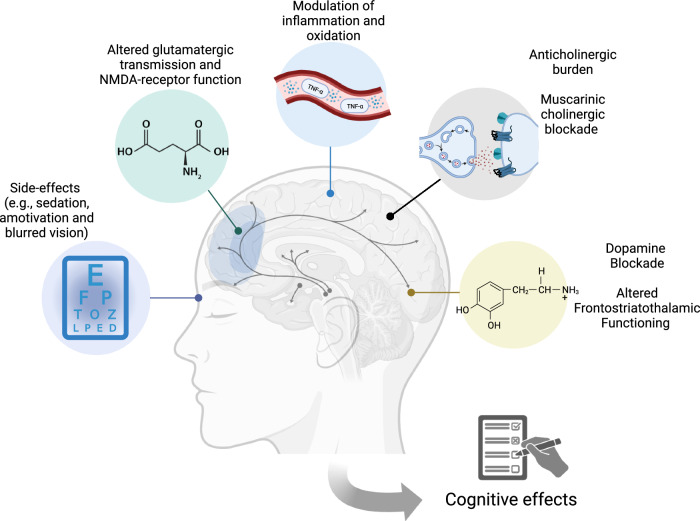
Potential mechanisms of the effects of antipsychotics on cognitive function.

We have proposed several potential mechanisms underlying the effects of antipsychotics on cognitive impairment (see Fig. 1), but the relative contributions of these mechanisms are likely to vary by antipsychotic class, dose, and duration, and require careful study to inform personalised care in the clinic. We call on the field to include more mechanistic aims and measures within dose-reduction, longitudinal, observational, and experimental studies to map and untangle the effect of these different mechanisms. These mechanistic aims should also be included in randomised controlled trials comparing antipsychotic dose reduction with maintenance. For example, including PET or Magnetic Resonance Spectroscopy measures to quantify neurotransmitter and neurometabolite levels, synthesis capacity, and receptor occupancy, or investigate associations between neuroimaging measures and normative maps of these molecular measures. Increasingly, functional neuroimaging (e.g., fMRI and EEG) is used to monitor longitudinal changes in brain connectivity and excitation/inhibition and indirectly infer balance. Similarly, including blood or saliva samples to index inflammatory response and estimated receptor occupancy markers, and the use of valid repeated anticholinergic burden and side-effect measures should be considered. Large datasets or clinical registries may also provide an avenue for understanding prescribing patterns and their association with cognitive and functional decline. Through focused investigation the field will be better able to model the precise mechanisms underlying the association between antipsychotics and cognitive function. This will advance personalised medicine and optimal treatment outcomes for people with psychosis.
